# Postpartum psychosis in bipolar disorder: no evidence of association with personality traits, cognitive style or affective temperaments

**DOI:** 10.1186/s12888-019-2392-0

**Published:** 2019-12-12

**Authors:** A. Perry, K. Gordon-Smith, I. Webb, E. Fone, A. Di Florio, N. Craddock, I. Jones, L. Jones

**Affiliations:** 1 Psychological Medicine, University of Worcester, Worcester, WR2 6AJ UK; 2 College of Medical and Dental Sciences, University of Birmingham, Birmingham, B15 2TT UK; 3 National Centre for Mental Health, Cardiff University, Cardiff, CF10 3XQ UK

**Keywords:** Postpartum psychosis, Bipolar disorder, Personality, Cognitive style, Temperament

## Abstract

**Background:**

Bipolar disorder has been associated with several personality traits, cognitive styles and affective temperaments. Women who have bipolar disorder are at increased risk of experiencing postpartum psychosis, however little research has investigated these traits and temperaments in relation to postpartum psychosis. The aim of this study is to establish whether aspects of personality, cognitive style and affective temperament that have been associated with bipolar disorder also confer vulnerability to postpartum psychosis over and above their known association with bipolar disorder.

**Methods:**

Personality traits (neuroticism, extraversion, schizotypy and impulsivity), cognitive styles (low self-esteem and dysfunctional attitudes) and affective temperaments (including cyclothymic and depressive temperaments) were compared between two groups of parous women with DSM-IV bipolar I disorder: i) 284 with a lifetime history of postpartum psychosis within 6 weeks of delivery (PP group), ii) 268 without any history of mood episodes with onset during pregnancy or within 6 months of delivery (no perinatal mood episode, No PME group).

**Results:**

After controlling for current mood state, and key demographic, clinical and pregnancy-related variables, there were no statistically significant differences between the PP and No PME groups on any of the personality, cognitive style or affective temperament measures.

**Conclusions:**

Personality traits, cognitive styles and affective temperaments previously shown to be associated with bipolar disorder in general were not specifically associated with the occurrence of postpartum psychosis. These factors may not be relevant for predicting risk of postpartum psychosis in women with bipolar disorder.

## Background

Postpartum psychosis (PP) is a severe psychiatric disorder, affecting 1–2 per 1000 births [31]. It is defined as an acute episode of mania or psychosis developing shortly after childbirth, typically within the first few weeks [13]. PP is considered a psychiatric emergency and requires hospitalisation in the majority of cases. Women classically present with frank psychosis, including hallucinations and delusions, mood lability, perplexity and confusion [13]. These symptoms develop rapidly, and vary dramatically from hour to hour [13], putting both the mother and, more rarely, baby at risk [27].

Women with bipolar disorder (BD) are at particularly high risk of developing PP; with episodes occurring in approximately 20% of deliveries to women with BD [49]. PP predominately affects women with a BD diathesis [13], with one study reporting that up to 95% of patients with PP satisfied Research Diagnostic Criteria for cyclic mood disorders at 5-year follow-up [51]. Family history of either PP or BD is a key risk factor for PP. Jones and Craddock [25] identified that women with BD and a first-degree relative with a history of PP had a 74% chance of developing PP themselves. Other potential risk factors for PP include primiparity [12], withdrawal of mood stabilising medication [48] and sleep deprivation [32]. Given the potential for adverse consequences associated with PP, it is important to identify other factors that put women with BD at high risk of experiencing PP.

Personality, cognitive style and affective temperaments have been investigated in relation to BD, but rarely in relation to PP [33]. Individuals with BD score higher than healthy controls on certain measures of affective temperament; most notably cyclothymic temperament [5, 15, 19, 35, 46] and depressive temperament [10, 11, 15, 35, 44]. Associations between specific personality traits and BD have also been demonstrated, such as higher levels of neuroticism [15, 17, 18, 20, 36], impulsivity [21, 38, 42, 47] and schizotypy [22] compared to healthy controls, and lower levels of extraversion [43, 45]. Furthermore, individuals with BD demonstrate distinct patterns of cognitive style compared to healthy controls, in particular lower self-esteem and higher levels of dysfunctional attitudes [28]. The nature of the association between BD, personality, cognitive style and affective temperaments remains unknown. Such traits may confer increased vulnerability to BD or alternatively, may be a consequence of the disorder. However, the relationship between personality and psychopathology is likely more complex; potentially being bidirectional, with the two aspects also sharing an underlying aetiology [30].

To date, only one study has specifically examined the relationship between personality factors and PP [33]. Using a prospective follow-up design, neuroticism assessed during pregnancy was not associated with PP among a mixed sample of women with BD and schizoaffective disorder. In contrast, higher levels of neuroticism were found to be associated with non-psychotic postpartum mood episodes among women with a history of mood disorders and in those without. However, neuroticism was the only personality factor examined, the sample size was small (12 women in the PP group) and comprised of diagnostically heterogeneous women. Thus, little is known about character traits that may confer vulnerability to PP over and above their known association with BD.

The aim of this study was to determine whether BD-related personality traits (neuroticism, extraversion, schizotypy and impulsivity), cognitive styles (low self-esteem and dysfunctional attitudes) and affective temperaments (including cyclothymic and depressive temperaments) were associated with PP in parous women with BD. The research has potential implications for improving understanding of the aetiology of PP and BD, as well as identifying women with BD at high risk of PP.

## Methods

### Participants

Participants were recruited by the Bipolar Disorder Research Network (BDRN; bdrn.org) as part of a large ongoing UK study into the genetic and non-genetic causes of mood disorders. The study has UK National Health Service (NHS) Research Ethics Committee approval and local Research and Development approval in all participating NHS Trusts/Health Boards. Informed consent was obtained from each participant. Participants were recruited systematically, via community mental health teams from across the UK, and non-systematically, via local and national media coverage and via advertisements placed in local general practitioner surgeries, on the BDRN website and circulated by the national charity, Bipolar UK.

Participants are included in the BDRN research programme if they meet the following inclusion criteria: 1) aged 18 years or older; 2) able to provide voluntary written informed consent; 3) are of UK White ethnicity, due to a focus on molecular genetics and 4) meet DSM-IV [3] criteria for major affective disorder. Individuals are excluded if they: 1) have only experienced affective illness in relation to or as a result of alcohol or substance misuse; 2) have only experienced affective illness as a result of medical illness or medication; 3) have an organic neurological or other cognitive impairment, which limits their ability to complete the assessments; or 4) are biologically related to another study participant.

Parous women with a best-estimate main lifetime diagnosis of DSM-IV bipolar I disorder (BD-I, recruited between 06/2001–03/15) who had completed at least one of the questionnaires listed below were included in the current study and stratified to two groups according to their lifetime perinatal psychiatric history:

I. PP group - women who had experienced an episode of mania or psychosis within 6 weeks of delivery (*n* = 284). As there is currently no consensus regarding the temporal cut-off that should be used to define the postpartum period, we used a definition of 6 weeks to be consistent with our previous studies and both DSM-5 (4 weeks) and ICD-11 [52] (6 weeks) postpartum onset criteria.
II. No perinatal mood episode (No PME) group – parous women without a history of mood episodes with onset during pregnancy or within 6 months following delivery (*n* = 268).

### Psychiatric assessment

Lifetime psychopathology was assessed via interview by a trained member of BDRN (research psychologist or psychiatrist) using the Schedules for Clinical Assessment in Psychiatry (SCAN, [50]). All participants were asked about the lifetime occurrence of pregnancies and lifetime occurrence of psychiatric episodes in the perinatal period. Where available, psychiatric case notes were also reviewed. Interview and case note data were combined for each participant to make key lifetime clinical and diagnostic ratings. In cases of doubt, diagnostic and clinical ratings were made by at least two members of the research team blind to each other’s ratings and consensus was reached via discussion where necessary. Inter-rater reliability was formally assessed using 20 random cases. Mean kappa statistics were 0.85 for DSM–IV diagnosis, 0.97 for lifetime perinatal psychiatric history, and ranged between 0.81 and 0.99 for other key clinical categorical variables. Mean intra-class correlation coefficients were between 0.91 and 0.97 for key clinical continuous variables (for example, age at illness onset).

### Questionnaires

Participants were asked to complete a battery of self-report questionnaires, either at the time of the initial clinical interview or subsequently as part of a questionnaire mail out.

As questionnaires were administered at different stages of the recruitment process, and completion optional, not all participants completed all questionnaires.

Six widely-used self-report questionnaires, all with demonstrated validity and reliability, were used in this study based on their measurements of personality traits, cognitive styles and affective temperaments that have previously been associated with BD.

1. Eysenck Personality Questionnaire (EPQ)

The 90-item version of the EPQ [16] was used in this study. Each item is rated ‘yes’ or ‘no’ by respondents, resulting in scores for three personality dimensions: extraversion (EPQ-E), neuroticism (EPQ-N) and psychoticism (EPQ-P). Only EPQ-E and EPQ-N were considered in this study due to their previous association with BD. Scores for EPQ-E range from 0 to 21 and EPQ-N from 0 to 23. Higher scores indicate higher levels of extraversion and neuroticism respectively.

2. Kings Schizotypy Questionnaire (KSQ)

The KSQ [29] is a 63-item questionnaire, which measures schizotypal personality traits on 7 subscales: recurrent illusions 1, social isolation, social anxiety, magical thinking, recurrent illusions 2, paranoid ideation and ideas of reference. Each item is rated ‘yes’ or ‘no’ by respondents. Total scores range from 0 to 63 and subscale scores from 0 to 9. Higher total and subscale scores indicate higher levels of schizotypy.

3. Barratt Impulsiveness Scale (BIS)

The BIS [37] is a 30-item questionnaire that measures trait impulsivity. Items are rated from 1 (absent) to 4 (most extreme). Total scores range from 30 to 120. Higher scores indicate higher levels of impulsivity.

4. Rosenberg Self-Esteem Questionnaire (SEQ)

The SEQ [41] is a 10-item questionnaire, which measures trait self-esteem. 5 questions are positively phrased and 5 questions are negatively phrased, corresponding to a positive and negative subscale respectively. Items are rated from 1 (strongly agree) to 4 (strongly disagree). Total scores range from 10 to 40, with higher scores indicating higher levels of self-esteem. Subscale scores range from 5 to 20, with high scores on the positive subscale indicating high positive self-esteem and high scores on the negative subscale indicating low negative self-esteem.

5. Dysfunctional Attitude Scale (DAS)

The DAS [40] measures underlying pervasive dysfunctional beliefs and attitudes. The 24 items are rated from 1 (totally agree) to 7 (totally disagree). Total scores range from 24 to 168. Three subscales are also scored (achievement, dependence, self-control), ranging from 0 to 56. Higher scores indicate a higher level of dysfunctional attitudes.

6. Temperament Evaluation of Memphis, Pisa, Paris and San Diego Auto-questionnaire Version (TEMPS-A)

TEMPS-A [1] is a 39-item questionnaire, which measures affective temperament on 5 subscales: cyclothymic, hyperthymic, depressive, irritable and anxious. TEMPS-A was developed specifically for use in an affectively ill population. Each item is rated ‘true’ or ‘false’ by respondents. With the exception of cyclothymic and anxious temperaments (scored from 0 to 12 and 0–3 respectively), subscale scores range from 0 to 8. Higher scores indicate higher affinity for each temperament.

### Measures of current mood state

Responses to the personality, cognitive style and affective temperament questionnaires can be affected by current mood symptoms. Therefore two widely used self-report measures of current mood symptoms, the Beck Depression Inventory (BDI) and the Altman Mania Scale (AMS), were administered alongside all questionnaires.

The BDI [4] is a 21-item questionnaire measuring the severity of current depression symptoms. Total scores range from 0 to 63. Higher scores indicate greater severity of depression.

The AMS [2] is a 5-item questionnaire measuring the severity of current manic symptoms. Total scores range from 0 to 20. Higher scores indicate greater severity of mania.

### Statistical analysis

Statistical analyses were carried out using the Statistical Package for the Social Sciences (SPSS) version 24.0. Categorical data (including demographic, clinical and pregnancy-related variables) were compared between the PP and No PME groups using chi-squared tests. Continuous data were not normally distributed; therefore medians, interquartile ranges and ranges are used to describe these data. Continuous data (including all questionnaire and subscale scores) were compared between the two groups using Mann-Whitney U tests. A stringent level of significance was set at 1% for the personality, cognitive style and affective temperament questionnaires to account for multiple testing.

Binary logistic regression analyses, using the enter method, were conducted to determine if any personality, cognitive style and affective temperament measures predicted group membership (PP versus No PME) controlling for potential demographic and clinical confounders (method of recruitment, age at interview, highest educational attainment and age at illness onset) and current mood state (BDI and AMS scores).

## Results

### Sample characteristics

There were significant differences in key demographic variables between the two groups (see Table 1). In the PP group, significantly more participants were recruited non-systematically than in the No PME group (75% vs. 59%, *p* < 0.001). Women in the PP group were significantly younger at the time of interview (median age 47 vs. 53 years, p < 0.001) and more likely to have completed higher education (46% vs. 36%, *p* = 0.014) compared to women in the No PME group. There were no significant differences between the groups for highest lifetime occupation and marital status.

**Table 1:** Comparison of demographic variables between the PP and No PME groups

	PP (*n* = 284)^1^	No PME (*n* = 268)^1^	PP versus No PME	
			χ^2^ or z-score	*P*-value
Method of recruitment, n (%)				
Systematic	71 (25.4)	109 (41.0)		
Non-systematic	208 (74.6)	157 (59.0)	14.85	< 0.001
Age at interview, years				
Median	47	53		
IQR (range)	15 (21–79)	16 (24–76)	−5.13	< 0.001
Highest educational attainment, n (%)				
No higher education	143 (53.6)	168 (64.1)		
Higher education	124 (46.4)	94 (35.9)	6.10	0.014
Highest occupation, n (%)				
Professional	157 (56.7)	124 (48.3)		
Non-Professional	115 (41.5)	128 (49.8)	3.83	0.15
Never worked	5 (1.8)	5 (1.9)		
Marital history, n (%)				
Married/lived as married	274 (96.8)	256 (95.9)		
Never married/lived as married	9 (3.2)	11 (4.1)	0.35	0.56

^1^Ns vary due to missing data. PP: postpartum psychosis. No PME: No perinatal mood episode

Lifetime clinical and pregnancy-related variables of the two groups are summarised in Table 2. Women in the PP group were significantly younger at illness onset (defined as age at first impairing episode of BD) than women in the No PME group (median age 22 vs. 30 years, *p* < 0.001). There were no significant differences between the groups for number of lifetime episodes of mania, number of lifetime episodes of depression, number of pregnancies and number of deliveries. Of women in the PP group, 45% had experienced the onset of their first impairing episode of BD during the postpartum period.

**Table 2:** Comparison of clinical, pregnancy-related and current mood variables between the PP and No PME groups

	PP (*n* = 284)^1^	No PME (*n* = 268)^1^	PP versus No PME	
			Z-score	*P*-value
Age at illness onset, years				
Median	22	30		
IQR (range)	10 (9–39)	19 (7–68)	−6.86	< 0.001
Number of episodes of mania				
Median	5	4		
IQR (range)	7 (1–100)	5 (1–100)	−1.63	0.10
Number of episodes of depression				
Median	5	5		
IQR (range)	8 (0–100)	8 (0–100)	−0.35	0.73
Number of pregnancies				
Median	2	2	−0.35	0.73
IQR (range)	1 (1–8)	1 (1–11)		
Number of deliveries				
Median	2	2		
IQR (range)	1 (1–6)	1 (1–8)	−1.23	0.22
First episode postpartum, n (%)				
Yes	124 (44.9)	–		
No	152 (55.1)	–	–	–
Beck Depression Inventory Score				
Median	7	9		
IQR (range)	13 (0–50)	15 (0–55)	−1.51	0.130
Altman Mania Scale score				
Median	2	3		
IQR (range)	4 (0–18)	5 (0–16)	−2.87	0.004

^1^Ns vary due to missing data. PP: postpartum psychosis. No PME: No perinatal mood episode

As shown in Table 2, AMS scores were significantly higher in the No PME group compared with the PP group (median score 3 vs. 2, *p* = 0.004). BDI scores did not significantly differ between the two groups.

### Comparison of personality, cognitive style and affective temperaments between the PP and no PME groups

Median total and subscale scores for the two groups on each of the personality, cognitive style and affective temperament measures are presented in Table 3. No significant differences were observed between the PP and No PME groups on any questionnaire measure, with the exception of KSQ magical thinking, for which scores were significantly lower in the PP group compared to the No PME group (1 vs. 2, *p* = 0.003). However, this relationship no longer remained significant after controlling for potential confounders (Table 3). Associations between all other questionnaire measures and postpartum psychiatric outcome remained non-significant in multivariate models.

**Table 3:** Personality, cognitive style and affective temperament measures in the PP and No PME groups

	PP (*n* = 284)	No PME (*n* = 268)	PP versus No PME	
			Unadjusted z-score (*p*-value)	Adjusted OR^a^(95% CI, *p*-value)
Eysenck Personality Questionnaire (EPQ)				
EPQ-Extraversion				
Median	12	11		
IQR (range)	8 (0–21)	10 (0–21)	−0.60 (0.55)	1.00 (0.97–1.04, 0.92)
EPQ-Neuroticism				
Median	15	15		
IQR (range)	9 (0–23)	9 (0–23)	0.47 (0.47)	1.00 (0.95–1.03, 0.48)
Kings Schizotypy Questionnaire				
Total score				
Median	15	16		
IQR (range)	15 (1–51)	17 (1–57)	−1.09 (0.28)	0.99 (0.97–1.04, 0.92)
Recurrent illusions 1				
Median	1	1		
IQR (range)	3 (0–9)	3 (0–9)	−0.30 (0.76)	0.95 (0.84–1.08, 0.45)
Social Isolation				
Median	3	3		
IQR (range)	4 (0–9)	3 (0–9)	−1.64 (0.10)	0.97 (0.87–1.10, 0.66)
Social anxiety				
Median	4	4		
IQR (range)	3 (0–9)	3 (0–9)	−0.49 (0.63)	1.10 (0.97–1.24, 0.16)
Magical thinking				
Median	1	2		
IQR (range)	2 (0–9)	2 (0–9)	−2.94 (0.003)	0.91 (0.80–1.04, 0.17)
Recurrent illusions 2				
Median	2	2		
IQR (range)	3 (0–9)	3 (0–9)	−0.036 (0.97)	1.03 (0.91–1.15, 0.68)
Paranoid ideation				
Median	1	1		
IQR (range)	2 (0–9)	2 (0–9)	−0.90 (0.37)	0.99 (0.85–1.14, 0.85)
Ideas of reference				
Median	2	2		
IQR (range)	3 (0–9)	3 (0–9)	−0.36 (0.72)	0.96 (0.86–1.07, 0.46)
Barratt Impulsiveness Scale				
Total score				
Median	63	64.5		
IQR (range)	15 (44–106)	17 (39–99)	−0.83 (0.41)	0.97 (0.95–1.00, 0.05)
Rosenberg Self-Esteem Inventory				
Total score				
Median	30	28.5		
IQR (range)	8 (12–40)	9 (13–40)	−0.82 (0.41)	1.03 (0.97–1.10, 0.31)
Positive subscale				
Median	15	15		
IQR (range)	4 (6–20)	4 (5–20)	−1.11 (0.27)	1.07 (0.94–1.22, 0.29)
Negative subscale				
Median	13	13.5		
IQR (range)	6 (5–20)	5 (5–20)	−0.39 (0.70)	1.05 (0.94–1.18, 0.38)
Dysfunctional Attitudes Scale				
Total score				
Median	90.5	86		
IQR (range)	29 (46–152)	29 (40–157)	−0.52 (0.60)	1.01 (0.99–1.02, 0.52)
Achievement				
Median	28.5	27		
IQR (range)	16 (8–56)	15 (7–56)	−0.52 (0.60)	1.01 (0.97–1.05, 0.66)
Dependency				
Median	32	30		
IQR (range)	11 (10–56)	13 (12–53)	−0.66 (0.51)	1.02 (0.98–1.07, 0.31)
Self-control				
Median	29	29		
IQR (range)	9 (16–43)	13 (12–54)	−0.38 (0.71)	1.00 (0.95–1.06, 0.94)
Temperament Evaluation of Memphis, Pisa, Paris and San Diego				
Cyclothymic				
Median	5	6		
IQR (range)	8 (0–12)	8 (0–12)	−1.02 (0.28)	0.97 (0.91–1.04, 0.42)
Depressive				
Median	1	1		
IQR (range)	3 (0–8)	4 (0–8)	−1.34 (0.18)	0.94 (0.83–1.07, 0.38)
Irritable				
Median	1	1		
IQR (range)	3 (0–8)	3 (0–8)	−1.08 (0.28)	1.00 (0.88–1.14, 1.00)
Hyperthymic				
Median	3	3		
IQR (range)	4 (0–8)	4 (0–8)	−0.60 (0.55)	0.95 (0.86–1.04, 0.26)
Anxious				
Median	1	1		
IQR (range)	2 (0–3)	2 (0–3)	−1.12 (0.27)	1.00 (0.82–1.23, 0.99)

^a^ Odds Ratios adjusted for method of recruitment, age at interview, highest educational attainment, age at illness onset, BDI score and AMS score. PP: postpartum psychosis. No PME: No perinatal mood episode

## Discussion

This study was the first to compare a range of personality traits, cognitive styles and affective temperaments between parous women with BD-I with and without a history of PP. No personality, cognitive style or affective temperament characteristics were identified that differentiated the two groups. Median scores for each of the questionnaire measures were remarkably similar between the two groups, showing little, if any, variation. The findings therefore suggest that these psychological traits, which have in previous literature been associated with the BD diathesis more generally, are not associated with the onset of PP specifically.

These findings are consistent with evidence implicating other, predominantly biological factors in the triggering of PP early in the postpartum. While the aetiology of PP remains poorly understood and is undoubtedly complex and multifactorial, previous studies have consistently found no association between PP and psychosocial factors, such as childhood trauma and other stressful life events [9, 14, 34, 39]**.** Together with the data reported here, this supports a key role for underlying neurobiological mechanisms**.** For example there is strong evidence to suggest that a specific vulnerability to the postpartum triggering of affective psychosis in BD is familial [23, 24], and therefore probably genetic. Jones and Craddock [23] reported that women with BD and a family history of PP are at a six-fold greater risk of suffering an episode of PP than parous women with BD and no family history of PP; this equates to 570 episodes of PP per 1000 deliveries. While molecular genetic studies have yet to provide a definitive answer, evidence from an initial linkage study has indicated the long arm of chromosome 16 as a possible location of a susceptibility gene [26].

The temporal relationship of PP with childbirth further implicates biological factors in the triggering of these episodes. Potential biological mechanisms may be hormonal, inflammatory or immunological [13]. A recent study has identified significant differences in inflammatory cell markers in the postpartum period between women with first-onset PP and healthy controls [6]. Furthermore, PP has also been associated with an increased incidence of autoimmune thyroid disease compared to healthy controls at both 4 weeks and 9 months postpartum [7]. Though hormone levels between women experiencing postpartum affective episodes do not appear to differ from healthy controls [8], there is evidence to suggest that some women with BD may be particular sensitive to the fluctuations in hormones that occur in relation to reproductive cycle events [27]. Thus, it remains likely that hormones play an important role in the pathophysiology of PP.

We have previously shown that postpartum depression is not associated with specific personality traits (neuroticism, extraversion and psychoticism) or cognitive styles (low self-esteem and dysfunctional attitudes) over and above their association with major recurrent depression, when a control group of parous women without postnatal depression was included in a similar study design to that used here [28]. Together with the findings reported here, our work supports the argument that while these personality, cognitive style and affective temperament characteristics are associated with vulnerability to affective illness in general, they do not influence the triggering of postpartum episodes specifically, at either end of the affective disorder spectrum.

### Strengths and limitations

This study has a number of strengths. Importantly the sample size was large and the groups well-defined and well-characterised. Detailed clinical data were gathered using gold standard semi-structured interviews and supplemented where available with psychiatric case notes. Furthermore, we were able to control for current mood state at the time personality, cognitive style and temperament was assessed.

Nevertheless**,** a number of limitations must be considered when interpreting the results. First, we investigated limited aspects of personality, cognitive style and affective temperament. Other aspects that may be associated with PP should be investigated in future research, for example, attachment styles and cognitive styles and beliefs related specifically to motherhood. Secondly, participants who were recruited both systematically (via NHS psychiatric services) and non-systematically (via advertisements) were included in analyses. However, method of recruitment was controlled for in multivariate models. Furthermore, we repeated analyses within systematically recruited participants only and the pattern of results remained unchanged. Thirdly, self-report measures were used for the assessment of personality, cognitive style and affective temperament. Such measures can be subjective and introduce the possibility of responder bias, however as discussed, potential current mood bias was adjusted for. More accurate results may be produced if self-report scales are used in combination with objective investigator-rated scales in future.

## Conclusion

PP is a serious psychiatric disorder which has potentially severe, adverse consequences for both mother and child. It is therefore vital to continue to work towards understanding the underlying aetiology and risk factors of PP. This study, which considered a large group of parous women with BD-I who have experienced PP and a control group of parous women with BD-I who have not experienced PP, suggests that aspects of personality, cognitive style and affective temperament known to be associated with BD in general do not influence vulnerability to PP specifically. These factors may not be relevant for predicting risk of PP in women with BD. Some women who experience PP may benefit from reassurance that aspects of their personality and temperament are unlikely to have an important role in the onset of the disorder.

## Data Availability

The datasets generated and/or analysed during the current study are not publicly available due to confidentiality but are available from the corresponding author on reasonable request.

## Abbreviations

AMS: Altman Mania Scale
BD: Bipolar disorder
BDI: Beck Depression Inventory
BD-I: Bipolar I disorder
BDRN: Bipolar Disorder Research Network
BIS: Barratt Impulsiveness Scale
DAS: Dysfunctional Attitude Scale
DSM-5: Diagnostic and Statistical Manual of Mental Disorders (5th edition)
DSM-IV: Diagnostic and Statistical Manual of Mental Disorders (4th edition)
EPQ: Eysenck Personality Questionnaire
EPQ-E: Eysenck Personality Questionnaire – Extroversion
EPQ-N: Eysenck Personality Questionnaire – Neuroticism
EPQ-P: Eysenck Personality Questionnaire – Psychoticism
ICD-11: International Classification of Diseases 11th Revision
KSQ: Kings Schizotypy Questionnaire
NHS: National Health Service
No PME: No perinatal mood episode
PP: Postpartum psychosis
SCAN: Schedules for Clinical Assessment in Psychiatry
SEQ: Rosenberg Self-Esteem Questionnaire
SPSS: Statistical Package for the Social Sciences
TEMPS-A: Temperament Evaluation of Memphis, Pisa, Paris and San Diego Auto-questionnaire Version

## References

[1] Akiskal HS, Akiskal KK, Haykal RF, Manning JS, Connor PD (2005). TEMPS-A: progress towards validation of a self-rated clinical version of the temperament evaluation of the Memphis, Pisa, Paris, and San Diego autoquestionnaire. J Affect Disord.

[2] Altman EG, Hedeker D, Peterson JL, Davis JM (1997). The altman self-rating mania scale. Biol Psychiatry.

[3] American Psychiatric Association. Diagnostic and Statistical Manual of Mental Disorders. Fourth Edi. Washington, DC: American Psychiatric Association; 2000.

[4] Beck AT, Steer RA, Brown GK (1996). Manual for the Beck depression inventory-II.

[5] Benazzi F, Akiskal HS (2005). A downscaled practical measure of mood lability as a screening tool for bipolar II. J Affect Disord.

[6] Bergink V, Burgerhout KM, Weigelt K, Pop VJ, de Wit H, Drexhage RC (2013). Immune system Dysregulation in first-onset postpartum psychosis. Biol Psychiatry.

[7] Bergink V, Kushner SA, Pop V, Kuijpens H (2011). Lambregtse-van Den berg MP, Drexhage RC, et al. prevalence of autoimmune thyroid dysfunction in postpartum psychosis. Br. J. Psychiatry.

[8] Bloch Miki, Daly Robert C, Rubinow David R (2003). Endocrine factors in the etiology of postpartum depression. Comprehensive Psychiatry.

[9] Brockington IF, Martin C, Brown GW, Goldberg D, Margison F (1990). Stress and puerperal psychosis. Br J Psychiatry.

[10] Cassano GB, Akiskal HS, Savino M, Musetti L, Perugi G (1992). Proposed subtypes of bipolar II and related disorders: with hypomanic episodes (or cyclothymia) and with hyperthymic temperament. J Affect Disord.

[11] Di Florio A, Hamshere M, Forty L, Green EK, Grozeva D, Jones I (2010). Affective temperaments across the bipolar-unipolar spectrum: examination of the TEMPS-A in 927 patients and controls. J Affect Disord.

[12] Di Florio A, Jones L, Forty L, Gordon-Smith K, Robertson Blackmore E, Heron J (2014). Mood disorders and parity - a clue to the aetiology of the postpartum trigger. J Affect Disord.

[13] Di Florio A, Smith S, Jones I (2013). Postpartum psychosis. Obstet Gynaecol.

[14] Dowlatshahi D, Paykel ES (1990). Life events and social stress in puerperal psychoses: absence of effect. Psychol Med.

[15] Evans L, Akiskal HS, Keck PE, McElroy SL, Sadovnick AD, Remick RA (2005). Familiality of temperament in bipolar disorder: support for a genetic spectrum. J Affect Disord.

[16] Eysenck HJ, Eysenck SB (1975). Manual for the Eysenck personality questionnaire. (EPQ-R adult).

[17] Fletcher K, Parker G, Barrett M, Synnott H, McCraw S (2012). Temperament and personality in bipolar II disorder. J Affect Disord.

[18] Gale CR, Hagenaars SP, Davies G, Hill WD, Liewald DCM, Cullen B (2016). Pleiotropy between neuroticism and physical and mental health: findings from 108 038 men and women in UK biobank. Transl Psychiatry.

[19] Hantouche EG, Akiskal HS, Lancrenon S, Allilaire JF, Sechter D, Azorin JM (1998). Systematic clinical methodology for validating bipolar-II disorder: data in mid-stream from a french national multi-site study (EPIDEP). J Affect Disord.

[20] Hayes JF, Osborn DPJ, Lewis G, Dalman C, Lundin A (2017). Association of Late Adolescent Personality with Risk for subsequent serious mental illness among men in a Swedish Nationwide cohort study. JAMA Psychiat.

[21] Henna E, Hatch JP, Nicoletti M, Swann AC, Zunta-Soares G, Soares JC (2013). Is impulsivity a common trait in bipolar and unipolar disorders?. Bipolar Disord.

[22] Heron J, Jones I, Williams J, Owen MJ, Craddock N, Jones LA (2003). Self-reported schizotypy and bipolar disorder: demonstration of a lack of specificity of the kings Schizotypy questionnaire. Schizophr Res.

[23] Jones I, Craddock N (2001). Familiality of the puerperal trigger in bipolar disorder: results of a family study. Am J Psychiatry.

[24] Jones I, Craddock N (2002). Do puerperal psychotic episodes identify a more familial subtype of bipolar disorder? Results of a family history study. Psychiatr Genet.

[25] Jones I, Craddock N (2005). Bipolar disorder and childbirth: the importance of recognising risk. Br J Psychiatry.

[26] Jones I, Hamshere M, Nangle JM, Bennett P, Green E, Heron J (2007). Bipolar affective puerperal psychosis: genome-wide significant evidence for linkage to chromosome 16. Am J Psychiatry.

[27] Jones I, Smith S (2009). Puerperal psychosis: identifying and caring for women at risk. Adv Psychiatr Treat.

[28] Jones L, Scott J, Cooper C, Forty L, Smith KG, Sham P (2010). Cognitive style, personality and vulnerability to postnatal depression. Br J Psychiatry.

[29] Jones LA, Cardno AG, Murphy KC, Sanders RD, Gray MY, McCarthy G (2000). The kings Schizotypy questionnaire as a quantitative measure of schizophrenia liability. Schizophr Res.

[30] Klein MH, Wonderlich S, Shea MT (1993). Models of relationships between personality and depression: Toward a framework for theory and research. Personal.

[31] Langan Martin J, McLean G, Cantwell R, Smith DJ (2016). Admission to psychiatric hospital in the early and late postpartum periods: Scottish national linkage study. BMJ Open.

[32] Lewis KJS, Di Florio A, Forty L, Gordon-Smith K, Perry A, Craddock N (2018). Mania triggered by sleep loss and risk of postpartum psychosis in women with bipolar disorder. J Affect Disord.

[33] Marks MN, Wieck A, Checkley SA, Kumar R (1992). Contribution of psychological and social factors to psychotic and non-psychotic relapse after childbirth in women with previous histories of affective disorder. J Affect Disord.

[34] McNeil TF, Persson-Blennow I, Binett B, Harty B, Karyd U (1988). A prospective study of postpartum psychoses in a high-risk group: 7. Relationship to later offspring characteristics. Acta Psychiatr. Scand.

[35] Mendlowicz MV, Jean-Louis G, Kelsoe JR, Akiskal HS (2005). A comparison of recovered bipolar patients, healthy relatives of bipolar probands, and normal controls using the short TEMPS-A. J Affect Disord.

[36] Nowakowska C, Strong CM, Santosa CM, Wang PW, Ketter TA (2005). Temperamental commonalities and differences in euthymic mood disorder patients, creative controls, and healthy controls. J Affect Disord.

[37] Patton JH, Stanford MS, Barratt ES (1995). Factor structure of the barratt impulsiveness scale. J Clin Psychol.

[38] Peluso MAM, Hatch JP, Glahn DC, Monkul ES, Sanches M, Najt P (2007). Trait impulsivity in patients with mood disorders. J Affect Disord.

[39] Perry A, Gordon-Smith K, Di Florio A, Forty L, Craddock N, Jones L (2016). Adverse childhood life events and postpartum psychosis in bipolar disorder. J Affect Disord.

[40] Power MJ, Katz R, McGuffin P, Duggan CF, Lam D, Beck AT (1994). The dysfunctional attitude scale (DAS). A comparison of forms a and B and proposals for a new subscaled version. J Res Pers.

[41] Rosenberg M (1965). Society and the adolescent self-image.

[42] Sanches M, Scott-Gurnell K, Patel A, Caetano SC, Zunta-Soares GB, Hatch JP (2014). Impulsivity in children and adolescents with mood disorders and unaffected offspring of bipolar parents. Compr Psychiatry.

[43] Sariusz-Skapska M, Czabala C, Dudek D, Zieba A, Rduch S (2003). Personality traits in patients with unipolar and bipolar disorder. Psychiatr Pol.

[44] Savitz J, van der Merwe L, Ramesar R (2008). Dysthymic and anxiety-related personality traits in bipolar spectrum illness. J Affect Disord.

[45] Smillie LD, Bhairo Y, Gray J, Gunasinghe C, Elkin A, McGuffin P (2009). Personality and the bipolar spectrum: normative and classification data for the Eysenck personality questionnaire-revised. Compr Psychiatry.

[46] Solmi M, Zaninotto L, Toffanin T, Veronese N, Lin K, Stubbs B (2016). A comparative meta-analysis of TEMPS scores across mood disorder patients, their first-degree relatives, healthy controls, and other psychiatric disorders. J Affect Disord.

[47] Swann AC, Anderson JC, Dougherty DM, Moeller FG (2001). Measurement of inter-episode impulsivity in bipolar disorder. Psychiatry Res.

[48] Viguera AC, Nonacs R, Cohen LS, Tondo L, Murray A, Baldessarini RJ (2000). Risk of recurrence of bipolar disorder in pregnant and nonpregnant women after discontinuing lithium maintenance. Am J Psychiatry.

[49] Wesseloo R, Kamperman AM, Munk-Olsen T, Pop VJM, Kushner SA, Bergink V (2016). Risk of postpartum relapse in bipolar disorder and postpartum psychosis: a systematic review and meta-analysis. Am J Psychiatry.

[50] Wing J. K. (1990). SCAN. Archives of General Psychiatry.

[51] Wisner KL, Peindl KS, Hanusa BH (1995). Psychiatric episodes in women with young children. J Affect Disord.

[52] World Health Organization (1993). The ICD-10 Classification of Mental and Behavioural Disorders.

